# Normative values of the vibration perception thresholds at finger pulps and metatarsal heads in healthy adults

**DOI:** 10.1371/journal.pone.0249461

**Published:** 2021-04-06

**Authors:** Linnéa Ekman, Eero Lindholm, Elisabeth Brogren, Lars B. Dahlin

**Affiliations:** 1 Department of Translational Medicine, Hand Surgery, Lund University, Malmö, Sweden; 2 Department of Clinical Sciences, Endocrinology, Lund University, Malmö, Sweden; 3 Department of Hand Surgery, Skåne University Hospital, Malmö, Sweden; University of Würzburg, GERMANY

## Abstract

**Aims:**

To establish normative values of vibration perception thresholds (VPTs), using multi-frequency vibrometry at finger pulps and at metatarsal heads of the foot in healthy adults. We also aimed to investigate factors that could potentially affect VPTs such as age, sex, height, weight, foot- or handedness and skin temperature.

**Methods:**

VPTs were examined in 924 healthy and randomly selected subjects in the southern Sweden (mean 46 years; 628 women and 296 men). Inclusion criterias were adult subjects (>18 years) in considerable health without diabetes mellitus or other nerve affecting disorders. VPTs were measured at the finger pulps of index and little finger, as well as the first and fifth metatarsal heads of the foot, through multi-frequency vibrometry using the VibroSense Meter® I device. Patient characteristics were recorded and skin temperature was measured before assessment of VPTs.

**Results:**

We present normative values of VPTs for a large population of both male and female subjects in various ages. VPTs detoriated as age increased (0.09–0.59 dB per year; p<0.001), i.e. progressing with normal aging. Increasing skin temperature affected VPTs in finger pulps, but not at metatarsal heads, with -0.2 to -1.6 dB, i.e. vibration perception improved with higher temperatures. Height was only found to affect the VPTs of metatarsal heads (250 Hz: 0.42 dB per cm). Sex, weight and handedness did not affect the VPTs.

**Conclusion:**

We investigated the normative values of VPTs and presented affecting factors as age, skin temperature and height. With these results, VPT testing through multi-frequency vibrometry is enabled to be used in a clinical practice as a diagnostic tool when investigating neuropathy and other neurological disorders.

## Introduction

Tactile perception in hands and feet is vital for our ability to explore textures, to perform delicate tasks through fine motor control, as well as for feedback from our feet for body spatial positioning and maintenance of balance and gait [1–6]. Feedback from tactile areas to the central nervous system depends on multiple afferent fibers and cutaneous receptors, among which the Meissner and Pacinian corpuscles are responsible for the detection of vibration stimulus. Meissner’s corpuscles respond to vibratory stimuli between 5 and 50 Hz, whereas the Pacinian corpuscles detects higher frequencies and are the most sensitive at 250 Hz [7–9].

Vibration perception threshold (VPT), i.e. the lowest vibrational intensity possible to perceive a vibration stimuli, is known to be impaired at an early stage in different neuropathies [10–14]. VPTs can be improved by enhancing the metabolic control of HbA_1c_ in patients with type 1 and type 2 diabetes [15–17]. Hence, examination of the VPTs can be useful both to detect neuropathy or other nerve disorders at an early stage, thus enabling preventive care and to monitor disease progression and treatment response [13,15]. However, normative data from a reference population is needed in order to compare VPTs from patients with suspected or confirmed neuropathies and other nerve disorders to a baseline distribution of VPTs. VPTs can be assessed for single frequencies by a tuning fork (128 Hz) or by a biothesiometer (100 Hz). In order to examine a larger spectrum of thresholds at tactile areas, as the cutaneous receptors detects different frequencies and supplies separate nerves, the technique of multi-frequency vibrometry is applied. Multi-frequency vibrometry has recently shown strong correlation with the results of nerve conduction studies (NCS), which is considered gold standard in examining nerve function and potential neuropathies [18,19]. Since NCS is more time-consuming and requires more personnel resources, it is more expensive and less accessible in comparison to the assessment of VPTs. In this study, we examined VPTs with a VibroSense Meter® (VibroSense Dynamics AB, Malmö, Sweden) where vibrations are applied through a probe vibrating at different frequencies (4, 8, 16, 32, 64, 125, 250 and 500 Hz).

Normative values of VPTs, through multi-frequency vibrometry in finger pulps and metatarsal heads have previously been published for children and adolescents [20] but for finger pulps in adults, the normative values have only been reported for a smaller population of 171 men [21]. However, normative values of VPT assessment through multi-frequency vibrometry for both finger pulps and metatarsal heads in a larger population of adults have never been established. Nor has the effect of influencing factors to specific frequencies been examined. As VPT testing could constitute a useful method applicable for examining and potentially diagnosing incipient neuropathies, it triggered the urge of establishing the baseline distribution of normative VPT values. A deeper understanding of factors that potentially could affect VPTs, is also needed in order to introduce VPT testing as a key tool in clinical praxis. Age and skin temperature have been presented as influencing factors to VPTs when investigated through a biothesiometer [22,23]. In a recent study, where a subset of data from the present study was used, VPTs have been investigated at metatarsal heads and shown to be affected by height and age [10]. Moreover, weight as well as hand- and footedness may potentially influence the VPTs [24–26]. Hence, we aimed to investigate and determine the vibration perception thresholds, at both finger pulps and metatarsal heads, in a wide range of frequencies in a broader study population of healthy adults and to investigate the influence of age, sex, height, weight, foot- or handedness and skin temperature.

## Methods and materials

### Ethical approval

The local ethics committee at Lund University approved the study (386/2007). The study was conducted in accordance with the Declaration of Helsinki and written informed consent was obtained from all participating subjects.

### Study population

In total, 924 participating subjects between 18 and 90 years (mean 46 years; 628 women and 296 men) were recruited consecutively during 3.4 years, from November 2014 to April 2018. Inclusion criteria were adult subjects, 18 years or above, in considerable health without diabetes mellitus or neurological disorders. Subjects were recruited in the southern of Sweden, particularly in Scania with its representative population, and consecutively included by invitation to various organizations and workplaces. For example, among subjects were care workers, engineers, students and university associates, all at various professional levels. Out of the 924 subjects examined, 11 participants were excluded due to various nerve affecting conditions; multiple sclerosis (n = 1), spinal stenosis (n = 2), chemotherapy treatment (n = 3) and ongoing medical investigation of nerve disease (n = 1), or due to incapacity of completing the examination (unable to follow instructions and unreliable results, n = 4). Hence, VPT measurements from 913 participating subjects (mean 46 years; 620 women, 293 men) were included for further analyses. Additionally, single measurements or frequencies were excluded if not meeting the criteria of a normal threshold curve, i.e. a structured VPT curve without outliers, or if skin temperature were below 20 and above 37°C [11,20,21,27]. Normal VPT curves have been more thoroughly described in Ising et al 2018 [11].

Subjects were inquired to complete a questionnaire regarding height, weight, date of birth as well as handedness (834 right- and 65 left-handed; 14 ambidextrous) and footedness (798 right- and 60 left-footed; 55 ambipedal). The questionnaire also comprised specific questions related to subjective neurological or vascular symtoms in hands and feet, to obtain information about potential nerve damage or dysfunction. Subjects with relevant neurological disorders were excluded.

### Multi-frequency vibrometry

VPTs were examined in the pulp of the index and little fingers of the right hand, as well as at the first and fifth metatarsal heads in the sole of the right foot, with multi-frequency tactilometry, using the VibroSense Meter® I device (VibroSense Dynamics AB, Malmö, Sweden). The investigated area was excited with a vibrating probe, carrying out vibrations at seven different frequencies (8, 16, 32, 64, 125, 250 and 500 Hz for finger pulps; 4, 8, 16, 32, 64, 125 and 250 Hz for metatarsal heads). The examination is fully automated and the frequencies are running from low to high. Vibrations were applied through the probe according to a von Békésy up and down psychophysical algorithm and the acceleration of the probe is expressed in decibels (dB; relative 10^−6^ m/s^2^). Acceleration started at 100 dB and increased with an amplitude ramp rate of 3 dB/s, until the subject perceived vibration and pressed down a response button. When pressed down, the intensity decreased in a corresponding speed of 3 dB/s, until the subject no longer perceived vibration and the button was released. Examinations of finger pulps were performed in accordance to ISO13091-1, Method A; without a surround and with a contact force of 0.15 ± 0.09 N between finger pulp and the probe [28]. This corresponds to a static skin intendent of approximately 1.5 mm. A continuously monitoring was made by the operator throughout the test to maintain the force within required limits [28]. VPT measurements at first and fifth metatarsal heads were performed in a similar manner with the VibroSense Meter® for feet, i.e. a modified hand device, which has been previously described in detail [20,29]. Since no standardization is published for examinations at metatarsal heads, a modification of the method was applied [11,20]. The examination procedure was, however, identical to the finger pulps concerning contact force. The probes of both devices measured 4 mm in diameter. All tests were controlled and stored through the VSM software (version 1.46.6; proprietary software) at a connected PC. VPTs are presented as a curve, vibrogram, which have been thoroughly described elsewhere [11,29]. All vibrograms were visually inspected by the operator, as no tracking algorithm is included in the software.

### Procedure of examining VPTs

Prior to assessment of VPTs, finger pulp temperatures were measured by an internal temperature probe of the VibroSense Meter® I. Temperatures of the surrounding area (20–22°C according to the requirements of ISO 13091–1) and at the metatarsal heads of the foot were measured with a hand-held thermometer [28]. All examinations were performed in an isolated and quiet room to exclude disturbing elements, and the subject was provided with hearing protection. Subjects were seated comfortably with the area to be examined, i.e. finger pulp or metatarsal head, placed on the probe. No visual contact with the probe was possible since the hand device was covered with a shield, and the foot per se covered the probe during the metatarsal examination. See Dahlin et al 2015 for more details [20]. All tests were performed by the same three examiners, two research nurses and one researcher, following the same protocol. The operator explained the procedure and encouraged the subject to keep a high level of concentration throughout the test, which lasted for approximately 4 minutes per finger pulp or metatarsal head. Subjects were instructed to press and keep the response button when perceiving vibrations and to release when no perception of vibration remained. This procedure was repeated four times at each frequency. Each examination, at the hand or foot, was preceded by a test recording at 16 Hz to acquaint the subject to the procedure. Every second subject was examined at hand at first, wheras the following subject was first examined at foot. However, VPT tests in hand were always initiated with index finger and in the foot at the first metatarsal. VPTs were calculated as mean values of the three last perception thresholds of each frequency, as the first precepted vibration was discarded according to ISO 13091–1 [28]. The frequency of 250 Hz at feet was first introduced to the test in September 2016 and hence, data are missing for this frequency in subjects examined before this date (n = 485).

### Statistical analyses

VPTs are presented for each finger pulp or metatarsal head as mean (95% confidence interval) and divided into groups by sex and age. Student’s paired samples *t*-test were performed to investigate mean differences (95% confidence interval) between the VPTs of index and little fingers, first and fifth metatarsal heads, index finger and first metatarsal as well as little finger and fifth metatarsal, separately. Correlations were determined by Pearson correlation matrix (rho >0.3; i.e. moderate and strong correlations, and p<0.001). To assess the effect of potentially affecting factors on VPTs, simple and multiple linear regression analyses were performed for age, sex, height, weight, foot- and handedness as well as skin temperature. Values are expressed in dB as unstandardized β-values with 95% confidence interval and as coefficients of determination (R^2^). P-values <0.001 were considered statistically significant (adjusted alpha-level value due to multiple comparisons). Statistical analyses were made using IBM SPSS Statistics (Statistical Package for the Social Sciencies, SPSS Inc., Chicago, Il, USA) version 25 for Mac.

## Results

### VPTs in finger pulps of the index and little fingers

**Fig 1:**
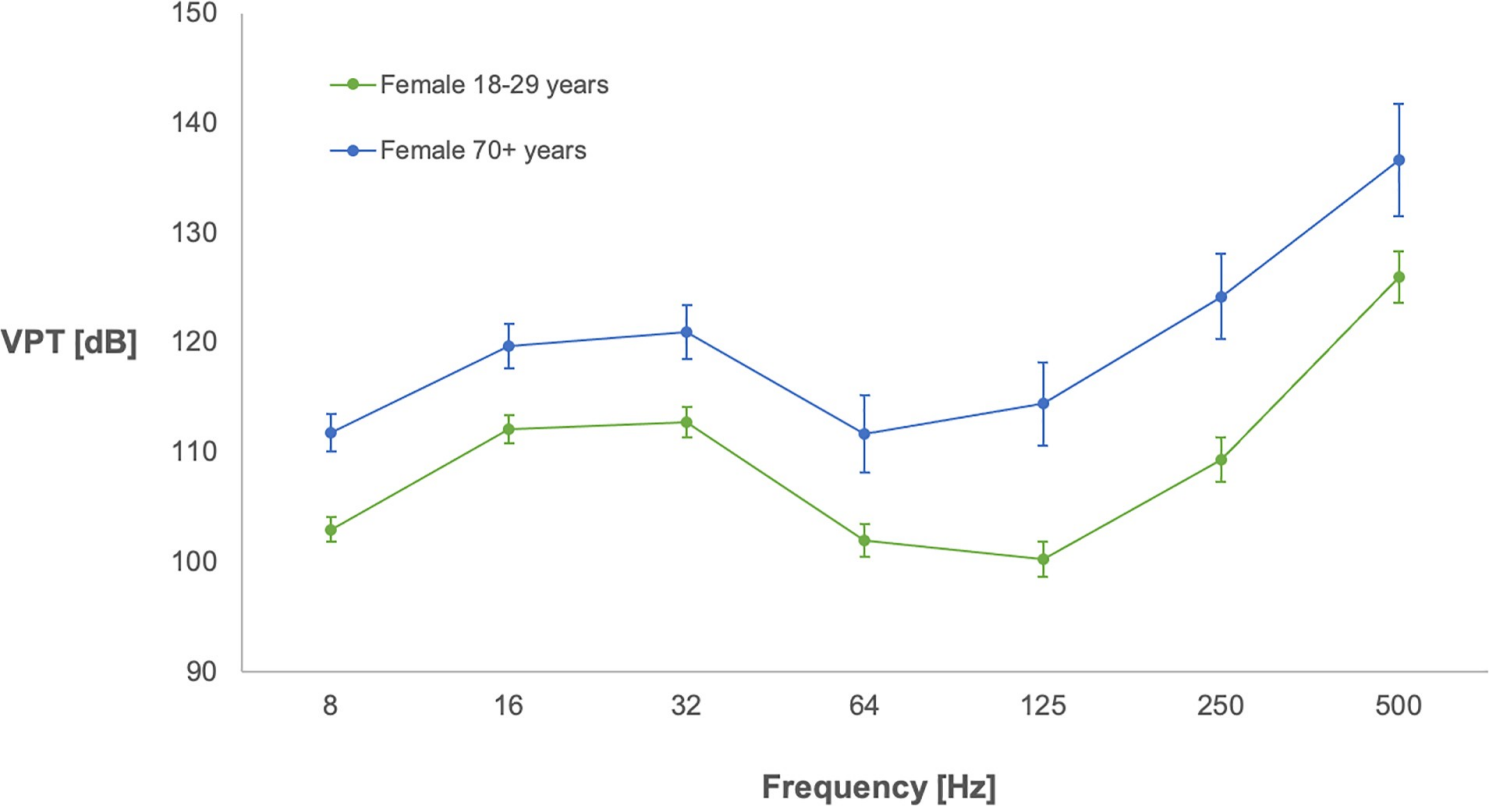
Vibration perception thresholds for index finger measured in females of two age groups. Y-axis shows mean vibration perception threshold (VPT) values in decibels (dB: relative 10^−6^ m/s^2^), and 95% confidence intervals, for the seven different frequencies (Hz) presented in the X axis. Subjects were females between 18 and 29 years (n = 58), as well as females over 70 years (n = 30).

VPTs in index and little fingers showed a pattern of increasing thresholds at higher frequencies, but with a decline at the 64 and 125 Hz frequencies (Fig 1). Thresholds increased with age in both male and female subjects for both fingers (Fig 1). Mean values for VPTs, divided in the sexes and decennial age groups, are presented for index (Table 1) and little fingers (Table 2), separately.

**Table 1:** Vibration perception thresholds in the finger pulp of the index finger at seven frequencies for 904 healthy adults.

Index finger									
Sex	Age (years)	Number	8 Hz	16 Hz	32 Hz	64 Hz	125 Hz	250 Hz	500 Hz
Male	18–29	58	103.5 (102–105)	113.5 (112–115)	111.9 (110–114)	100.7 (99–103)	100.5 (99–102)	108.8 (106–111)	124.2 (121–128)
	30–39	53	104.6 (103–106)	113.6 (112–115)	114.3 (113–116)	102.0 (100–104)	100.1 (98–102)	106.2 (104–109)	123.6 (121–126)
	40–49	75	105.7 (105–107)	113.9 (113–115)	113.5 (112–115)	102.3 (101–104)	102.9 (101–104)	112.0 (110–114)	127.6 (125–130)
	50–59	44	107.8 (106–109)	115.2 (113–117)	114.5 (112–117)	103.7 (102–106)	104.0 (102–106)	111.5 (109–114)	127.3 (124–130)
	60–69	32	108.0 (106–110)	116.8 (115–118)	117.8 (116–119)	106.0 (104–108)	108.5 (106–111)	118.6 (115–122)	134.1 (130–139)
	> 70	30	111.6 (109–114)	119.4 (118–121)	120.7 (118–123)	112.2 (109–115)	116.2 (113–119)	127.2 (124–131)	139.3 (134–145)
Female	18–29	97	103.0 (102–104)	112.1 (111–113)	112.7 (111–114)	102.0 (100–103)	100.3 (99–102)	109.3 (107–111)	126.0 (124–128)
	30–39	121	103.2 (102–104)	111.6 (111–113)	112.4 (111–114)	101.5 (100–103)	100.7 (99–102)	109.5 (108–111)	125.3 (123–127)
	40–49	140	104.3 (103–105)	114.2 (113–115)	112.1 (111–113)	101.6 (100–103)	102.8 (102–104)	112.8 (111–114)	127.8 (126–130)
	50–59	146	105.7 (105–106)	114.3 (113–115)	113.3 (112–114)	104.1 (103–105)	105.1 (104–106)	113.1 (112–115)	129.5 (128–131)
	60–69	78	107.3 (106–109)	115.4 (114–117)	115.0 (113–117)	105.6 (104–108)	107.0 (105–109)	114.8 (112–117)	129.2 (127–131)
	> 70	30	111.7 (110–113)	119.7 (118–122)	120.9 (118–123)	111.7 (108–115)	114.4 (111–118)	124.2 (120–128)	136.7 (132–142)

Values expressed in dB, as mean and 95% confidence interval.

**Table 2:** Vibration perception thresholds in the finger pulp of the little finger at seven frequencies for 903 healthy adults.

Little finger									
Sex	Age (years)	Number	8 Hz	16 Hz	32 Hz	64 Hz	125 Hz	250 Hz	500 Hz
Male	18–29	58	104.0 (103–105)	111.1 (110–113)	115.2 (113–117)	103.6 (102–106)	101.4 (99–103)	107.4 (105–110)	126.3 (123–130)
	30–39	53	104.8 (104–106)	111.4 (110–113)	115.3 (114–117)	104.8 (103–107)	101.4 (100–103)	107.0 (105–109)	124.8 (122–127)
	40–49	75	104.4 (103–105)	111.7 (111–113)	114.9 (113–116)	105.7 (104–107)	103.8 (102–105)	112.2 (110–114)	128.9 (126–132)
	50–59	44	106.8 (105–108)	113.1 (111–115)	114.3 (112–117)	104.2 (102–106)	102.5 (100–105)	109.1 (107–111)	126.2 (123–129)
	60–69	32	108.4 (106–110)	114.9 (114–116)	119.4 (118–121)	110.0 (108–112)	109.7 (107–112)	118.3 (114–122)	(129–136)
	> 70	30	110.8 (109–113)	118.8 (117–121)	123.2 (121–125)	115.7 (113–118)	117.3 (114–121)	126.3 (122–130)	140.2 (135–146)
Female	18–29	97	102.6 (101–104)	109.9 (109–111)	114.2 (113–116)	103.9 (102–105)	101.7 (100–103)	108.6 (107–111)	127.2 (125–130)
	30–39	121	102.1 (101–103)	108.9 (108–110)	112.8 (112–114)	103.2 (102–104)	101.5 (100–103)	109.2 (108–111)	124.5 (122–127)
	40–49	139	103.6 (103–104)	110.9 (110–112)	113.9 (113–115)	103.7 (102–105)	102.9 (102–104)	111.7 (110–113)	127.1 (125–129)
	50–59	146	104.6 (104–105)	112.0 (111–113)	115.8 (115–117)	105.9 (105–107)	105.0 (104–106)	111.9 (110–113)	127.8 (126–129)
	60–69	78	106.8 (106–108)	113.9 (113–115)	116.0 (114–118)	108.4 (107–110)	108.1 (106–111)	115.1 (112–118)	128.9 (126–132)
	> 70	30	110.6 (109–112)	117.9 (116–120)	121.9 (120–124)	112.9 (109–116)	114.1 (110–118)	122.9 (120–126)	141.3 (137–146)

Values expressed in dB, as mean and 95% confidence interval.

Paired comparisons showed small mean differences between index and little fingers at the frequencies of 16, 32 and 64 Hz [8 Hz: 0.65 (0.2–1.1), p = 0.009; 16 Hz: 1.83 (1.3–2.4), p<0.001; 32 Hz: -1.96 (-2.5–-1.4), p<0.001; 64 Hz: -2.35 (-2.9–-1.8), p<0.001; 125 Hz: -0.67 (-1.2–-0.1), p = 0.020; 250 Hz: 0.04 (-0.6–0.7), p = 0.912; 500 Hz: -1.14 (-2.0–-0.3), p = 0.011]. Moderate positive correlations were found between the index and little finger pulps for all frequencies (p<0.001, *r*: 0.376–0.611; Table 3) [30].

**Table 3:** Pearson correlations between vibration perception thresholds in hands and feet for different frequencies in 892 healthy subjects.

	4 Hz	8 Hz	16 Hz	32 Hz	64 Hz	125 Hz	250 Hz	500 Hz
Index and little fingers	n/a	0.418	0.376	0.468	0.552	0.589	0.611	0.478
First and fifth metatarsal	0.439	0.514	0.555	0.595	0.667	0.667	0.767	n/a
Index finger and first metatarsal	n/a	-	-	-	0.321	0.399	0.303	n/a
Little finger and fifth metatarsal	n/a	0.393	0.418	0.378	0.444	0.531	0.498	n/a

Data are rho-values, only rho-values of >0.30 are presented (<0.30 indicated by -). All values showed p<0.0001.

n/a: not applicable.

### Linear regression analyses for VPTs in index and little fingers

Results from multiple regression of the frequencies of 8, 16, 250 and 500 Hz are presented in Table 4.

**Table 4:** Multiple regression analyses of VPT changes in low and high frequencies for five independent variables.

Frequency	Sex	Age	Height	Weight	Skin temperature	Footedness	R^2^
Index finger							
8 Hz	-	0.138 (0.12–0.16)	-	-	-0.253 (-0.36–-0.15)	n/a	0.185
16 Hz	-	0.110 (0.09–0.13)	-	-	-0.248 (-0.36–-0.14)	n/a	0.117
250 Hz	-	0.244 (0.21–0.28)	-	-	-1.236 (-1.42–-1.06)	n/a	0.288
500 Hz	-	0.204 (0.16–0.26)	-	-	-1.576 (-1.79–-1.36)	n/a	0.254
Little finger							
8 Hz	-	0.126 (0.10–0.15)	-	-	-	n/a	0.156
16 Hz	-	0.129 (0.10–0.16)	-	-	-	n/a	0.124
250 Hz	-	0.238 (0.20-0-28)	-	-	-1.158 (-1.34–-0.98)	n/a	0.260
500 Hz	-	0.186 (0.14–0.23)	-	-	-1.649 (-1.86–-1.44)	n/a	0.263
First metatarsal							
8 Hz	-	0.188 (0.15–0.23)	-	-	-	-	0.135
16 Hz	-	0.226 (0.19–0.27)	-	-	-	-	0.166
250 Hz	-	0.544 (0.47–0.62)	0.423 (0.22–0.63)	-	-	-	0.391
Fifth metatarsal							
8 Hz	-	0.144 (0.11–0.18)	-	-	-	-3.542 (-5.63–-1.46)	0.118
16 Hz	-	0.167 (0.13–0.21)	-	-	-	-	0.147
250 Hz	-7.071 (-10.30–-3.85)	0.486 (0.42–0.55)	0.418 (0.23–0.60)	-	-	-	0.418

Values are expressed in dB, if p<0.001, as unstandardized β-values with 95% confidence interval and as coefficient of determination (R^2^).

n/a: not applicable.

No correlations were found between handedness and VPTs (p>0.06) and thus, the variable was not employed in the multiple regression analysis. In the simple regression analyses, women had lower VPTs than men at the frequencies of 8 and 16 in the little finger (1.51–1.71 dB, p<0.001), but no difference between the sexes was found in the multiple regression. There was a linear positive relationship between age and VPTs in both finger pulps for all frequencies in the simple regression analysis (0.11–0.25 dB per year, p<0.001) which remained when the model was adjusted for age, sex, height, weight and skin temperature (0.11–0.24 dB per year; selected frequencies are presented in Table 4). At lower frequencies, height and weight were positively correlated to the VPTs when analysed by simple regression but the effect disappeared in the adjusted model. Higher skin temperatures were associated to lower VPTs in both finger pulps (-1.66–-0.26 dB per 1 degree Celsius, p<0.001). When adjusting the model for multiple variables, this relationship remained for all frequencies except for 8 and 16 Hz regarding the little finger (Table 4). Variance of VPTs showed similar patterns in index finger as in the little finger, with skin temperature and age as the major affecting variables and at a similar levels of explanation. The R^2^ coefficients are < 0.29 for all multiple regression analyses, indicating that the five independent variables could explain at maximum 29% of the variation in VPTs among the 913 subjects.

### VPTs in sole of the foot at the first and fifth metatarsal heads

**Fig 2:**
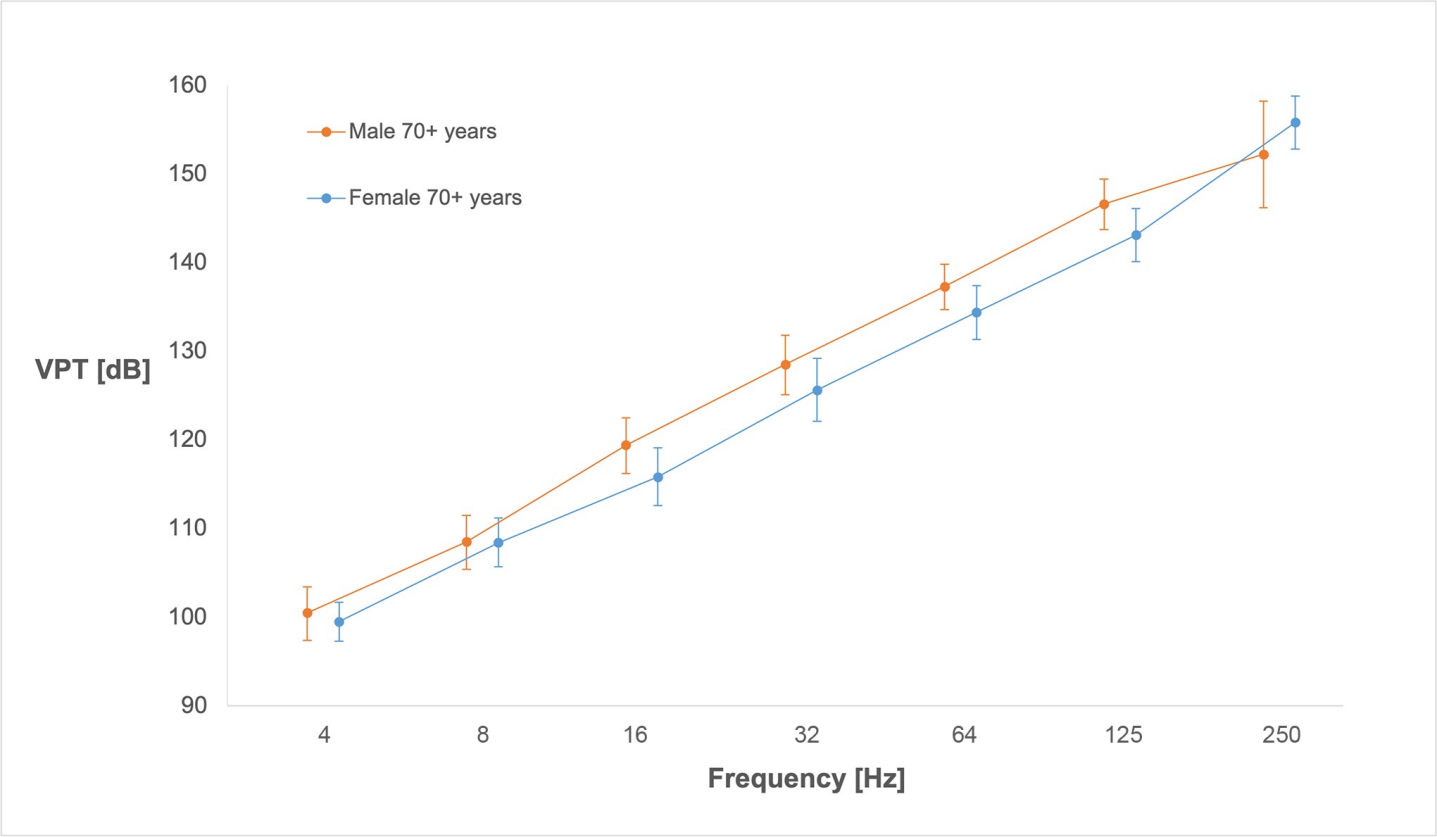
Vibration perception thresholds for fifth metatarsal head measured in males and females over 70 years. Y-axis shows mean vibration perception threshold (VPT) values in decibels (dB; relative 10^−6^ m/s^2^), and 95% confidence intervals, for the seven different frequencies (Hz) presented in the X axis. Subjects were males and females, all over 70 years (n = 29 in each group).

Assessment of VPTs at the first and fifth metatarsal heads did not show the same pattern as for finger pulps, i.e. with declining thresholds at 64 and 125 Hz. On the contrary, thresholds increased constantly with higher frequencies, as well as with age, in both male and female subjects (Fig 2). Mean values for VPTs, grouped by sex and decennial age groups, are presented for the first (Table 5) and fifth metatarsal heads (Table 6), separately.

**Table 5:** Vibration perception thresholds at the first metatarsal head in the sole of the foot at seven frequencies for 892 healthy adults.

First metatarsal head									
Sex	Age (years)	Number	4 Hz	8 Hz	16 Hz	32 Hz	64 Hz	125 Hz	250 Hz*
Male	18–29	57	94.1 (92–96)	98.4 (96–100)	105.2 (103–107)	111.5 (110–113)	113.6 (111–116)	113.4 (111–116)	124.6 (122–128)
	30–39	52	95.3 (94–97)	100.1 (98–102)	107.8 (105–110)	114.5 (112–117)	118.5 (116–121)	119.8 (117–123)	135.1 (131–139)
	40–49	75	95.5 (94–97)	102.1 (100–104)	109.5 (108–111)	116.8 (115–119)	124.0 (122–126)	126.6 (124–129)	136.3 (132–141)
	50–59	43	97.4 (95–99)	103.2 (101–106)	110.6 (108–113)	119.5 (117–122)	127.3 (124–130)	131.4 (127–136)	143.4 (135–152)
	60–69	31	98.3 (96–101)	106.7 (103–110)	115.7 (112–119)	123.4 (119–128)	132.9 (129–137)	140.5 (135–146)	152.6 (143–162)
	> 70	26	102.2 (100–105)	110.1 (107–113)	119.4 (116–123)	128.2 (125–132)	135.4 (132–139)	145.5 (142–149)	157.7 (153–162)
Female	18–29	95	92.9 (92–94)	99.1 (98–101)	105.9 (104–107)	111.9 (110–114)	113.0 (111–115)	113.8 (112–116)	128.7 (125–132)
	30–39	122	92.7 (92–94)	98.4 (97–100)	105.3 (104–107)	111.9 (110–113)	114.8 (113–116)	116.3 (114–118)	129.8 (126–134)
	40–49	141	94.8 (94–96)	99.9 (99–101)	108.0 (107–109)	115.0 (114–116)	119.4 (118–121)	121.6 (119–124)	137.5 (134–141)
	50–59	144	96.0 (95–97)	102.1 (101–103)	109.8 (108–111)	117.1 (116–119)	122.7 (121–125)	127.9 (126–130)	140.4 (136–144)
	60–69	77	97.2 (96–99)	104.7 (103–107)	112.5 (110–114)	119.5 (117–122)	126.7 (124–129)	131.7 (128–135)	146.0 (141–152)
	> 70	29	101.5 (99–104)	110.4 (107–113)	119.3 (116–123)	128.8 (126–132)	136.9 (133–141)	145.0 (141–149)	152.9 (149–156)

Values expressed in dB, as mean and 95% confidence interval.

*Data only available for 407 subjects due to addition of the 250 Hz frequency in September 2016.

**Table 6:** Vibration perception thresholds at the fifth metatarsal head in the sole of the foot at seven frequencies for 894 healthy adults.

Fifth metatarsal head									
Sex	Age (years)	Number	4 Hz	8 Hz	16 Hz	32 Hz	64 Hz	125 Hz	250 Hz*
Male	18–29	57	94.6 (93–96)	100.1 (98–102)	106.8 (105–109)	112.4 (110–114)	113.9 (111–116)	113.9 (111–117)	124.5 (121–128)
	30–39	52	97.4 (96–99)	102.5 (100–105)	110.4 (108–113)	115.7 (113–118)	119.1 (116–122)	119.9 (117–123)	135.7 (130–141)
	40–49	74	96.6 (95–98)	102.2 (100–104)	109.9 (108–112)	117.1 (115–119)	123.3 (121–125)	126.3 (124–129)	138.6 (135–142)
	50–59	43	97.7 (96–100)	103.7 (101–106)	112.1 (110–115)	120.2 (117–123)	127.2 (124–130)	130.5 (126–135)	140.6 (133–148)
	60–69	32	98.8 (96–102)	106.7 (103–110)	114.9 (111–119)	123.5 (120–128)	132.1 (128–136)	139.5 (135–144)	148.3 (141–155)
	> 70	29	100.5 (97–104)	108.5 (105–111)	119.4 (116–123)	128.5 (125–132)	137.3 (135–140)	146.6 (144–149)	152.2 (146–158)
Female	18–29	95	93.8 (93–95)	98.4 (97–100)	105.8 (104–107)	111.5 (110–113)	112.6 (111–115)	113.7 (111–116)	129.8 (127–133)
	30–39	121	92.8 (92–94)	98.6 (97–100)	105.5 (104–107)	112.5 (111–114)	115.1 (113–117)	116.9 (115–119)	131.4 (128–135)
	40–49	140	93.0 (92–94)	99.8 (98–101)	106.9 (105–108)	114.3 (113–116)	119.7 (118–121)	122.6 (121–124)	138.0 (135–141)
	50–59	145	94.7 (94–96)	101.6 (100–103)	109.5 (108–111)	117.2 (116–119)	123.8 (122–125)	127.1 (125–129)	141.4 (137–145)
	60–69	77	96.6 (95–98)	103.0 (101–105)	110.2 (108–113)	119.5 (117–122)	127.8 (125–130)	131.4 (128–135)	147.2 (144–150)
	> 70	29	99.5 (97–102)	108.4 (106–111)	115.8 (113–119)	125.6 (122–129)	134.4 (131–137)	143.1 (140–146)	155.8 (153–159)

Values expressed in dB, as mean and 95% confidence interval.

*Data only available for 409 subjects due to addition of the 250 Hz frequency in September 2016.

Paired comparisons showed no differences in VPTs between the first and fifth metatarsals. In addition, a variety of moderate and strong positive correlations were found between metatarsals for all frequencies (p<0.001, *r*: 0.439–0.767; Table 3).

### Linear regression analyses for VPTs in first and fifth metatarsal heads

Results from the multiple regression analyses are presented for the frequencies 8, 16 and 250 Hz in Table 4.

The effect of age was similar in both simple and multiple regression models, where VPTs increased with age for all frequencies (0.09–0.59 dB per year; p<0.001; selected frequencies for the multiple regression are presented in Table 4). In multiple regression analyses, height affected VPTs for frequencies between 64–250 Hz (0.31–0.47 dB per cm; p<0.001), whereas weight showed no effect (Table 4). Higher skin temperatures seemed to lower the VPT for the 125 Hz frequency only (MTI: -0.56 dB, MTV: -0.62 dB per 1 degree Celsius; p<0.001). In the adjusted model, men had lower VPTs at the fifth metatarsal head for the frequency of 250 Hz (-7.07 dB, p<0.001; Table 4). No correlation or effect could be found between footedness and VPTs measured at MTI (p>0.19) in the simple regression analysis and thus, the variable was not employed in the multiple regression model. However, linear regression analysis showed that patients who were left-footed had lower VPTs at MTV for the 8 Hz frequency (-3.79 dB, p<0.001) and this effect remained in the multiple regression as well (Table 4).

### Comparisons of VPTs at finger pulps and metatarsal heads

Differences with similar patterns were found between the index finger and the first metatarsal head [8 Hz: 3.8 (3.2–4.5); 16 Hz: 4.5 (3.8–5.2); 32 Hz: -2.8 (-3.6–-2.0); 64 Hz: -17.7 (-18.6–-16.9); 125 Hz: -20.7 (-21.6–-19.8); 250 Hz: -25.6 (-27.4–-23.9); p<0.001 for all], as between the little finger and the fifth metatarsal head [p<0.001; 8 Hz: 3.4 (2.9–3.9); 16 Hz: 2.9 (2.3–3.4); 32 Hz: -0.9 (-1.5–-0.3); 64 Hz: -15.9 (-16.6–-15.2); 125 Hz: -20.1 (-20.9–-19.4); 250 Hz: -25.6 (-26.9–-24.3); p<0.001 for all].

Moderate positive correlations were seen between index finger and first metatarsal head for higher frequencies (64, 125 and 250 Hz; *r*: 0.303–0.309, p<0.001) as well as between little finger and fifth metatarsal head for all frequencies (*r*: 0.378–0.531; Table 3).

## Discussion

With this study, we present a reference material of VPT values for investigation through multi-frequency vibrometry using the VibroSense Meter® I device. The study comprises examinations of VPTs in a large population of 924 healthy subjects, both male and female in ages between 18 and 90 years. As the methods of VPT testing are developed, the need for a greater knowledge about the normal physiology of our vibrotactile sense is growing. In order to relate VPT results to actual pathology, both arising and already existing, it is important to understand the impact of potential patient-related factors such as age, sex, biometry and skin temperature. The overall aim of understanding the physiology of VPTs, and its methods for investigation, is to achieve the ability to exert these testings into clinical practice.

The procedure of examining the index and little fingers through vibrometry mirrors the function of the median and ulnar nerves, and is therefore equivalent to the NCS examination of these. Investigation by NCS in the lower limb is carried out through the peroneal, sural and tibial nerves whereas VPT testing focuses on the medial and lateral metatarsals, which both are innervated by two branches of the tibial nerve. Thus, these areas are supported by different plantar branches, i.e. the lateral and medial plantar nerves, reflecting diverse plantar sensory areas which are important for the function of balance and gait [31]. Hence, examination of these areas can be considered to yield a more functional measure than NCS.

In our data, VPT curves showed a pattern with increasing thresholds with rising frequency, as well as a decrease in thresholds between 32 and 125 Hz at finger pulps, but not at metatarsal heads. This is in agreement with the patterns seen in children and adolescents as reported by Dahlin et al [20]. In fact, the patterns as well as differences and affecting factors within the younger subjects in our study were similar, although the authors in the previously published study did not intentionally investigate the predicting factors [20]. Age was the main affecting factor found in our study, where the impact on VPTs showed to be significant for all frequencies in all areas examined (0.09–0.59 dB per year; p<0.001). Hence, VPTs are increasing with age with up to 0.59 dB per year. This change is probably best explained by normal age-related neurobiological changes, such as degeneration of nerve fibers and changes of the mechanoreceptors responsible for vibration perception [32]. According to previous studies, palmar density of Pacinian corpuscles as well as the distal limb density of Meissner´s corpuscles, are decreasing with age [33,34].

In our study, we found skin temperature to be an important factor affecting the VPTs, where sensitivity decreased with lower temperature in the fingers. According to the ISO 13091–1, VPTs should not be significantly influenced by skin temperatures in the range of 27–35°C [28]. However, temperatures between 20–37°C were allowed in this present study. Therefore, we found that skin temperature affected VPTs with -0.2 to -1.6 dB per increased degree in all frequencies for the index finger, as well as with -1.2 to -1.6 degrees for the frequencies of 250 and 500 Hz for the little finger. Thus, low skin temperatures can affect the patients ability to perceive the vibrations and should therefore be avoided when examining finger pulps. In contrast, skin temperature did not influence the VPTs at metatarsal heads of the foot.

Overall, there was no substantial difference in VPTs between the sexes. The number of women was considerably higher than the number of male subjects in our study. This uneven distribution was a result of variation in recruitment, since a higher proportion of female dominated professions reported interest in participating in the study. We adjusted for sex in all the multiple regression analyses and only found lower VPTs for men at the frequency of 250 Hz in the fifth metatarsal head. However, one should bare in mind that this specific frequency was introduced to the metatarsal heads in the late phase of our study and thus not comprise an as large portion of subjects as the other frequencies. Additionally, after introducing the 250 Hz frequency to the foot device, our recruitment was focused on elder subjects in the ages of over 70 years as well as younger students in the ages of 20 to 25 years. According to previous studies, differences dependent on age are significant among elder subjects, but not among younger male and female subjects [35,36]. Additionally, the category of subjects over 70 years in our study is comprising a range of ages between 70 and 90 years. Hence, the results regarding the 250 Hz frequency should be carefully interpreted as the distribution between the sexes here could be rather misleading. Additionally, heat and cold thresholds measured in clinical practice should be interpreted with caution in patients at high age and such a thought could also be applied for the measurements of VPTs. Our findings that the normal values for VPTs are similar between men and women stands in contrast to previously published data that rather suggested that women present with greater sensitivity at higher frequencies [37].

We included both right and left dominant (hand-/footedness) subjects although all our examinations were performed on the right extremities. We could not see any differences in VPTs regarding dominance except for the frequency of 8 Hz examined at the fifth metatarsal head. Whether this was a coincidence or not is difficult to tell as the distribution of right- and left-footed subject was uneven. Additionally, several participants had difficulties in determining which foot was the dominant one. This could have been avoided if a questionnaire was applied [38]. Threshold patterns correlated between the index and litte fingers, as well as between the first and fifth metatarsals of the foot. These results refers to the unique patterns in hands and feet due to diverse density and function of the receptors responsible for vibration perception at both sites [6,39–41]. As expected, VPTs were higher at metatarsal heads than within the finger pulps. Additionally, subject height presented as an affecting factor to VPTs for the frequency of 250 Hz at the metatarsals, but not the finger pulps. This is in compliance with the length-dependent pattern of neuropathy, which is more pronounced in the lower extremities and increases with height [31,42]. Considering the effect of height at the higher frequencies, but not the lower ones, a possible explanation could be attributed to the different densities of Meissner and Pacinian corpuscles. According to Strzalkowski et al, the density of FAII afferents, terminating in Pacinian corpuscles, reaches less than half the density of the FAI afferents, associated to the Meissners corpuscles, in the sole of the foot [31]. However, in contrast to the previously mentioned article, we could not see any clear differences between the lateral and medial metatarsals regarding this. Other studies have shown that the total nerve fiber densities are distinctly lower in the legs, compared to the arms [41,43].

The potential factors that were investigated in our study, i.e. age, sex, height, footedness and skin temperature, only showed to represent a 29% level of explanation of VPT differences in finger pulps, whereas the same factors accounted for a 42% explanation rate at metatarsal heads. The remaining percents might be explained by anatomical variations, other external factors of impact or individual differences. For instance, the within-subjects variability is shown to be greater in elderly than within younger subjects [44,45]. Hence, investigations of further affecting variables are still needed.

### Limitations and strengths

Although the recruitment of study subjects was addressed to a variety of citizens, it resulted in a non-evenly distribution between men and women as well as an skewness in age distribution, were the majority of subjects were in the ages of 30 and 59 years. Difficulties in recruiting elderly were mainly due to concomitant illnesses or diseases, whereas the youngest subjects were uneasily motivated to participate. An important limitation to the investigational method of our study is that the VPT testing thorugh multi-frequency vibrometry demands an active participation of the examined subject. This is in contrast to the methods of nerve conduction studies which can be brought out passively. Accordingly, examination through vibrometry implies higher demands of cognition. Another limitation of the test method is that frequencies only are presented in a consecutive sequence from low to high. Despite these limitations, the method offers several benefits or advantages in comparison to the gold standard, as mentioned above. In comparison to NCS, VPT testing through multi-frequency vibrometry is less expensive and time-consuming. It is also more accessible, as it requires less professional time for examination and interpretation. VPT testing is a non-invasive method that also could be considered less unpleasant than NCS, as some patients experience it as uncomfortable. Clinical implications that follows our study are for example to facilitate follow-ups of nerve affecting disorders in both primary and diabetic care, as well as to constitute a useful tool in neuropathic investigations. Impact of repeated measurements has been evaluated and the results implicated that practice effects are non-existing when examining VPTs through vibrometry at an annual basis [46]. If applicable, the possibility to compare subjects to their own former results would therefore be an important tool in these investigations.

## Conclusion

Vibrotactile perception thresholds were examined in a large sample of healthy adult subjects of both sexes to compile a material of the normative values of VPTs in finger pulps and metatarsal heads. We found that VPTs deteriorate progressively as a part of the normal aging. Other major predicting factors found in our study was skin temperature in fingers, as well as height regarding the VPTs at metatarsal heads of the foot. These results enables the knowledge about the VPT pattern to be used in a clinical practice as a diagnostic tool when investigating neuropathy and other neurological disorders.

## References

[1] Yau JM, Kim SS, Thakur PH, Bensmaia SJ. Feeling form: the neural basis of haptic shape perception. J Neurophysiol 2016;115:631–42. 10.1152/jn.00598.2015 26581869 PMC4752307

[2] Fallon JB, Bent LR, McNulty PA, Macefield VG. Evidence for strong synaptic coupling between single tactile afferents from the sole of the foot and motorneurons supplying leg muscles. J Neurophysiol 2005;94:3795804. 10.1152/jn.00359.2005 16079197

[3] Zehr EP, Stein RB. What functions do reflexes serve during human locomotion? Prog Neurobiol 1999;58:185–205. 10.1016/s0301-0082(98)00081-1 10338359

[4] Collins DF, Refshauge KM, Todd G, Gandevia SC. Cutaneous receptors contribute to kinesthesia at the index finger, elbow and knee. J Neurophysiol 2005;94:1699–706. 10.1152/jn.00191.2005 15917323

[5] Aimonetti JM, Hospod V, Roll JP, Ribot-Ciscar E. Cutaneous afferents provide a neuronal population vector that encodes the orientation of human ankle movements. J Physiol 2007;580:649–58. 10.1113/jphysiol.2006.123075 17255169 PMC2075553

[6] Strzalkowski ND, Ali RA, Bent LR. The firing characteristics of foot sole cutaneous mechanoreceptor afferents in response to vibration stimuli. J Neurophysiol 2017;118:1931–42. 10.1152/jn.00647.2016 28679842 PMC5626905

[7] Bell J, Bolanowski S, Holmes MH. The structure and function of Pacinian corpuscles: a review. Prog Neurobiol 1994;42:79–128. 10.1016/0301-0082(94)90022-1 7480788

[8] Mountcastle VB, LaMotte RH, Carli G. Detection thresholds for stimuli in humans and monkeys: comparison with threshold events in mechanoreceptive afferent nerve fibers innervating the monkey hand. J Neurophysiol 1972;35:122–36. 10.1152/jn.1972.35.1.122 4621505

[9] LaMotte RH, Mountcastle VB. Capacities of humans and monkeys to discriminate vibratory stimuli of different frequency and amplitude: a correlation between neural events and physiological measurements. J Neurophysiol 1975;38:539–59. 10.1152/jn.1975.38.3.539 1127456

[10] Lindholm E, Löndahl M, Fagher K, Apelqvist J, Dahlin LB. Strong association between vibration perception thresholds at low frequencies (4 and 8 Hz), neuropathic symptoms and diabetic foot ulcers. PLoS ONE 2019;14(2):e0212921. 10.1371/journal.pone.0212921 30817797 PMC6394961

[11] Ising E, Dahlin LB, Elding Larsson H. Impaired vibrotactile sense in children and adolescents with type 1 diabetes–Signs of peripheral neuropathy. 2018;13(4):e0196243. 10.1371/journal.pone.0196243 PMC5908163 29672623

[12] Gerhardsson L, Gillström L, Hagberg M. Test-retest reliability of neurophysiological tests of hand-arm vibration syndrome in vibration exposed workers and unexposed referents. J Occup Med Toxicol 2014;9(1):38. 10.1186/s12995-014-0038-1 25400687 PMC4232643

[13] Thomsen NO, Cederlund R, Speidel T, Dahlin LB. Vibrotactile sense in patients with diabetes and carpal tunnel syndrome. Diabet Med 2011;28:1401–6. 10.1111/j.1464-5491.2011.03308.x 21480975

[14] Lundborg G, Lie-Stenström AK, Sollerman C, Strömberg T, Pyykö J. Digital vibrogram: a new diagnostic tool for sensory testing in compression neuropathy. J Hand Surg 1986;11A:693–9. 10.1016/s0363-5023(86)80014-4 3760497

[15] Dahlin LB, Elqzyri T, Löndahl M, Ekman L, Lindholm E. Improved metabolic control using glucose monitoring systems leads to improvement in vibration perception thresholds in type 1 diabetes patients. Acta Diabetol 2019; 10.1007/s00592-019-01450-2 31705298 PMC7093360

[16] Fujita Y, Fukushima M, Suzuki H, Taniguchi A, Nakai Y, Kuroe A, et al. Short-term intensive glycemic control improves vibratory sensation in type 2 diabetes. Diabetes Res Clin Pract. 2008;80(1):e16–9. 10.1016/j.diabres.2007.12.011 18262304

[17] Ishibashi F, Taniguchi M, Kosaka A, Uetake H, Tavakoli M. Improvement in neuropathy outcomes with normalizing HbA1c in patients with type 2 diabetes. Diabetes Care. 2019;42(1):110–8. 10.2337/dc18-1560 30455338

[18] Lindholm E, Ekman L, Apelqvist J, Löndahl M, Dahlin LB. 55^th^ EASD Annual Meeting of the European Association for the Study of Diabetes: Barcelona, Spain, 16–20 September 2019. Diabetologia. 2019;62(Suppl 1):S471–S471. 10.1007/s00125-019-4946-6 31384961

[19] Peterson M, Pingel R, Rolandsson O, Dahlin LB. Vibrotactile perception on the sole of the foot in an older group of people with normal glucose tolerance and type 2 diabetes. SAGE Open Medicine. 2020;8:1–9. 10.1177/2050312120931640 32587694 PMC7294473

[20] Dahlin LB, Güner N, Elding Larsson H, Speidel T. Vibrotactile perception in finger pulps and in the sole of the foot in healthy subjects among children and adolescents. PLoS One 2015; 10(3):e0119753. 10.1371/journal.pone.0119753 25835710 PMC4383580

[21] Lundström R, Strömberg T, Lundborg G. Vibrotactile perception threshold measurements for diagnosis of sensory neuropathy. Int Arch Occup Environ Health 1992;64:201–7. 10.1007/BF00380910 1328063

[22] Bloom S, Till S, Sönksen P, Smith S. Use of a biothesiometer to measure individual vibration thresholds and their variation in 519 non-diabetic subjects. Br Med J (Clin Res Ed). 1984;288(6433):1793–5. 10.1136/bmj.288.6433.1793 6428547 PMC1441832

[23] Meh D, Denislic M. Influence of age, temperature, sex, height and diazepam on vibration perception. J Neurol Sci. 1995;134(1–2):136–42. 10.1016/0022-510x(95)00230-9 8747856

[24] Thrainsdottir S, Malik RA, Rosén I, Jakobsson F, Bakhtadze E, Petersson J, et al. Sural nerve biopsy may predict future nerve dysfunction. Acta Neurol Scand. 2009;120(1):38–46. 10.1111/j.1600-0404.2008.01118.x 19154542

[25] Hage JJ, van der Steen LP, de Groot PJ. Difference in sensibility between the dominant and nondominant index finger as tested using the Semmes-Weinstein monofilaments pressure aesthesiometer. J Hand Surg Am. 1995;20(2):227–9. 10.1016/s0363-5023(05)80012-7 7775756

[26] Ricci PT. Possible interaction between vibration thresholds by sex and motor dominance in the index finger and big toe. Percept Mot Skills. 1997;85(3 Pt 1):1091–8. 10.2466/pms.1997.85.3.1091 9399324

[27] Stromberg T, Dahlin LB, Lundborg G. Vibrotactile sense in the hand-arm vibration syndrome. Scand J Work Environ Health 1998;24:495–502. 10.5271/sjweh.374 9988092

[28] ISO 13091–1. Mechanical vibration–Vibrotactile perception thresholds for the assessment of nerve dysfunction–Part 1: Methods of measurement at the fingertips. International Organization of Standardization, Geneva; 2011.

[29] Nelander J, Speidel T, Björkman A, Dahlin LB. Vibration thresholds are increased at low frequencies in the sole of the foot in diabetes–a novel multi-frequency approach. Diabet Med. 2012;29(12):e449–56. 10.1111/dme.12024 22998552

[30] Akoglu H. User’s guide to correlation coefficients. Turk J Emerg Med. 2018;18(3):91–3. 10.1016/j.tjem.2018.08.001 30191186 PMC6107969

[31] Strzalkowski NDJ, Peters RM, Inglis JT, Bent LR. Cutaneous afferent innervation of the human foot sole: what can we learn from single-unit recordings?. J Neurophysiol. 2018;120(3):1233–46. 10.1152/jn.00848.2017 29873612 PMC6171067

[32] Palve SS, Palve SB. Impact of aging on nerve conduction velocities and late responses in healthy individuals. J Neurosci Rural Pract. 2018;9(1):112–6. 10.4103/jnrp.jnrp_323_17 29456354 PMC5812134

[33] Bruce MF. The relation of tactile thresholds to histology in the fingers of elderly people. J Neurol Neurosurg Psychiatry. 1980;43(8):730–4. 10.1136/jnnp.43.8.730 7431035 PMC490646

[34] Iwasaki T, Goto N, Goto J, Ezure H, Moriyama H. The aging of human Meissner’s corpuscles as evidenced by parallel sectioning. Okajimas Folia Anat Jpn. 2003;79(6):185‐189. 10.2535/ofaj.79.185 12776944

[35] Venkatesan L, Barlow SM, Kieweg D. Age- and sex-related changes in vibrotactile sensitivity of hand and face in neurotypical adults. Somatosens Mot Res. 2015;32(1):44‐50. 10.3109/08990220.2014.958216 25248543

[36] Gescheider GA, Bolanowski SJ, Hall KL, Hoffman KE, Verrillo RT. The effects of aging on information-processing channels in the sense of touch: I. Absolute sensitivity. Somatosens Mot Res. 1994;11(4):345‐357. 10.3109/08990229409028878 7778411

[37] Gandhi MS, Sesek R, Tuckett R, Bamberg SJ. Progress in vibrotactile threshold evaluation techniques: a review. J Hand Ther. 2011;24(3):240‐256. 10.1016/j.jht.2011.01.001 21439781

[38] Elias LJ, Bryden MP, Bulman-Fleming MB. Footedness is a better predictor than is handedness of emotional lateralization. Neuropsychologia. 1998;36(1):37–43. 10.1016/s0028-3932(97)00107-3 9533385

[39] Mildren RL, Bent LR. Vibrotactile stimulation of fast-adapting cutaneous afferents from the foot modulates proprioception at the ankle joint. J Appl Physiol 2016;120(8):855–64. 10.1152/japplphysiol.00810.2015 26823342 PMC4835905

[40] Johansson RS, Vallbo ÅB. Tactile sensibility in the human hand: Relative and absolute density of four types of mechanoreceptive units in glabrous skin. J Physiol 1979;286:283–300. 10.1113/jphysiol.1979.sp012619 439026 PMC1281571

[41] Thomsen NO, Mojaddidi M, Malik RA, Dahlin LB. Biopsy of the posterior interosseous nerve: a low morbidity method for assessment of peripheral nerve disorders. Diabet Med. 2009;26(1):100‐104. 10.1111/j.1464-5491.2008.02629.x 19125770

[42] Sorensen L, Molyneaux L, Yue DK. Insensate versus painful diabetic neuropathy: the effects of height, gender, ethnicity and glycaemic control. Diabetes Res Clin Pract. 2002;57(1):45‐51. 10.1016/s0168-8227(02)00010-4 12007729

[43] O’Sullivan DJ, Swallow M. The fibre size and content of the radial and sural nerves. J Neurol Neurosurg Psychiatry. 1968;31(5):464‐470. 10.1136/jnnp.31.5.464 4303798 PMC496402

[44] Stevens JC, Cruz LA, Marks LE, Lakatos S. A multimodal assessment of sensory thresholds in aging. J Gerontol B Psychol Sci Soc Sci. 1998;53(4):263–72. 10.1093/geronb/53b.4.p263 9679518

[45] Iandolo R, Carè M, Shah VA, Schiavi S, Bommarito G, Boffa G, et al. A two alternative forced choice method for assessing vibrotactile discrimination thresholds in the lower limb. Somatosens Mot Res. 2019;36(2):162–70. 10.1080/08990220.2019.1632184 31267810 PMC6653623

[46] Ekman L, Persson Löfgren J, Dahlin LB. Examining practice effects in repeated measurements of vibration perception thresholds on finger pulps of healthy individuals—Is it possible to improve your results over a clinically relevant test interval?. PLoS One. 2019;14(12):e0226371. 10.1371/journal.pone.0226371 31846492 PMC6917284

